# Long-term outcomes in deceased versus living donor liver transplantation for hepatocellular carcinoma: a bi-institutional study of 486 cases

**DOI:** 10.1007/s00464-025-12011-w

**Published:** 2025-08-11

**Authors:** Fabrizio Di Benedetto, Cristiano Guidetti, Dimitri Aristotle Raptis, Gian Piero Guerrini, Paolo Magistri, Massimo Malagò, Stefano Di Sandro, Dieter Clemens Broering

**Affiliations:** 1 Hepato-Pancreato-Biliary Surgery and Liver Transplantation Unit, University Hospital of Modena “Policlinico”, University of Modena and Reggio Emilia, 41124 Modena, Italy; 2 Department of Liver Transplantation and Hepatobiliary Surgery, Organ Transplant Center of Excellence, King Faisal Specialist Hospital and Research Center, Riyadh, Saudi Arabia

**Keywords:** Living donor liver transplant, Hepatocellular carcinoma, Liver transplantation, Oncological outcomes, Living donation

## Abstract

**Background:**

Liver transplantation (LT) is a curative treatment for hepatocellular carcinoma (HCC), but access is often limited by organ shortage and prolonged waiting times. Living donor liver transplantation (LDLT) offers timely transplantation and may improve oncologic outcomes compared to deceased donor liver transplantation (DDLT).

**Methods:**

This retrospective cohort study included 486 patients with HCC who underwent LT at two high-volume centers between 2010 and 2020. Outcomes were compared between LDLT and DDLT recipients. Survival analyses were performed using Kaplan–Meier estimates and Cox regression models.

**Results:**

A total of 182 patients received LDLT and 304 received DDLT. Baseline tumor burden and liver function were comparable. LDLT was associated with shorter time to transplant (186 vs. 410 days, *p* < 0.001), fewer downstaging procedures, and improved survival. Five-year overall survival was 82% for LDLT versus 73% for DDLT (*p* = 0.010); disease-free survival was 93% versus 83% (*p* = 0.003). On multivariable analysis, DDLT (HR 3.03, *p* = 0.017) and BCLC B/C stage (HR 1.98, *p* = 0.017) were independent predictors of recurrence.

**Conclusion:**

LDLT is associated with superior long-term outcomes in patients with HCC, independent of tumor stage and timing. These findings support the broader use of LDLT as an effective oncologic strategy.

**Graphical abstract:**

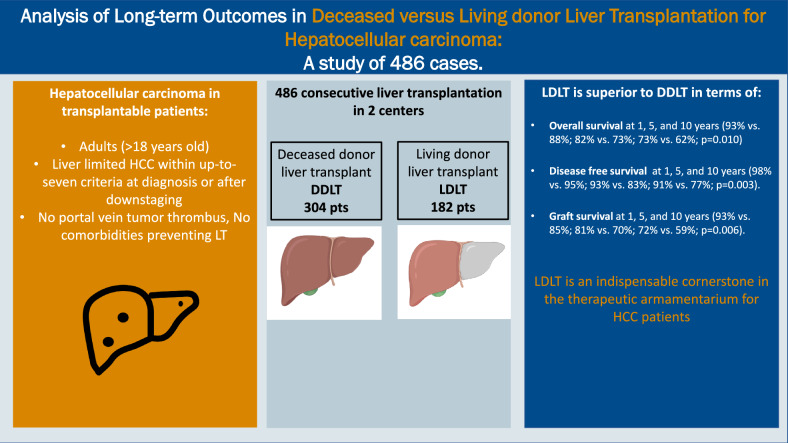

Hepatocellular carcinoma (HCC) is a major cause of cancer-related mortality worldwide, and its incidence continues to rise [1, 2]. Liver transplantation (LT), despite being constrained by organ scarcity, remains the most effective therapeutic option for selected patients with liver-limited disease burden [3, 4].

A critical limitation in the field of liver transplantation, however, is the persistent shortage of donor organs. This often results in prolonged waiting times and a significant risk of dropout due to tumor progression. As shown by Mehta et al., the probability of dropout can reach 25–30% within the first year on the waiting list, especially when transplantation is delayed beyond 6 months, leading to poorer post-transplant outcomes [5].

To address this challenge, various strategies have been implemented to expand the donor pool. These include the use of extended criteria donors (ECD) and donation after cardiac death (DCD).

Moreover, split liver transplant procedure was introduced to address the problem of organ shortage; however, it is limited by questionable results and low diffusion, especially in Europe [6].

In parallel, living donor liver transplantation (LDLT) has gained momentum, particularly in countries with limited access to deceased donors [7].

Over time, LT has become the treatment of choice for patients with HCC who meet appropriate selection criteria. As illustrated by Vitale et al., early LT, performed before biological progression, maximizes oncologic benefit without compromising long-term outcomes [8].

A steadily growing number of patients with hepatocellular carcinoma (HCC) are currently considered eligible for liver transplantation (LT). This trend, together with the progressive expansion of transplant indications, contributes to lengthening waiting lists and increasing the overall demand for donor organs [9].

In recent years, LDLT has emerged as a safe and effective strategy for managing patients with HCC. Several studies have reported better post-transplant survival and lower recurrence rates compared to deceased donor liver transplantation (DDLT) [9, 10].

Moreover, the introduction of minimally invasive techniques for donor hepatectomy may further improve donor safety and increase acceptance of LDLT programs [11].

Based on this background, our hypothesis is that LDLT enables timely liver transplantation within a multimodal and multidisciplinary framework, thus improving oncologic outcomes for patients with HCC. The primary aim of this study is to compare the long-term results of LDLT and DDLT in patients with HCC. A secondary objective is to evaluate the impact of transplant timing on overall and disease-free survival.

## Materials and methods

### Study design

This is a retrospective bi-institutional cohort study conducted at two high-volume liver transplant centers. The study was reported in accordance with the Strengthening the Reporting of Observational Studies in Epidemiology (STROBE) guidelines [12].

The study design was based on the PICOS framework:

- Participants (P): adult patients with HCC eligible for LT,
- Intervention (I): LDLT
- Comparator (C): DDLT,
- Outcomes (O): overall survival (OS), graft survival (GS), and recurrence-free survival (RFS).

The participating centers included the King Faisal Specialist Hospital and Research Center (KFSHRC), Riyadh, and the University Hospital of Modena (UNIMORE), Italy. KFSHRC performs approximately 300 LDLTs per year, while UNIMORE carries out around 150 LTs annually.

All patients provided written informed consent prior to LT. The study was approved by the local Institutional Review Board (IRB, protocol PDTA 10/24) and conducted in accordance with the Declaration of Helsinki and the Declaration of Istanbul.

The data used in this study are available upon request from the corresponding author and access is subject to approval by the IRB.

### Patient selection

All adult patients undergoing LT for HCC between January 1, 2010, and December 31, 2020, were included in the study.

Eligibility was discussed within multidisciplinary tumor boards at both institutions. While the up-to-seven criteria were commonly used as a guiding framework to assess tumor burden (at presentation or after downstaging), final decisions regarding transplant eligibility were made individually by each institutional committee, based on tumor response, biological behavior, and overall clinical condition [13].

In Modena, all patients were listed for DDLT. In Riyadh, LDLT was considered in the presence of a suitable donor, with DDLT used as an alternative in their absence.

During the waiting period, patients underwent standardized follow-up every 3 months, which included liver function tests, alpha-fetoprotein levels, contrast-enhanced abdominal CT or MRI, and chest imaging. Bridging and downstaging therapies were applied according to institutional protocols and shared clinical principles. Immediate pre-transplant reassessment was performed using the same diagnostic work-up to confirm tumor status and transplant eligibility.

Patients were excluded if they presented with macroscopic portal vein tumor thrombosis, evidence of extrahepatic metastases, or comorbidities that contraindicated safe LT.

Exceptionally, three patients with type 2 portal vein tumor thrombus at diagnosis underwent successful downstaging with transarterial radioembolization (TARE) and were reassessed as within criteria prior to transplant as described by other groups in literature [14].

To reduce selection bias, all eligible patients were included consecutively.

### Data collection

Data were extracted from prospectively maintained institutional databases and included demographic, clinical, and oncologic variables. Comorbidities were evaluated using the Charlson Comorbidity Index (CCI) [15].

Tumor-related data encompassed number and size of nodules, vascular invasion, alpha-fetoprotein (AFP) levels at diagnosis and at the time of transplant, and classification according to the Milan criteria [16], up-to-seven criteria [17], and BCLC staging [18].

Treatment strategies prior to transplantation, including resection, percutaneous ethanol injection, radiofrequency or microwave ablations, transarterial chemoembolization (TACE), and transarterial radioembolization (TARE), were recorded.

Radiologic response to treatment was evaluated when applicable using modified RECIST (mRECIST) criteria [19].

Intraoperative variables included operative time (OT), estimated blood loss (EBL), and the administration of blood products such as packed red blood cells (pRBC), fresh frozen plasma (FFP), and platelets.

The occurrence of reperfusion syndrome was recorded according to the definition by Siniscalchi and colleagues [20]. Technical aspects of the transplant procedure, such as the method of caval and biliary reconstruction and use of arterial conduits, were also documented.

Postoperative outcomes included laboratory parameters on postoperative day five (AST, ALT, INR, creatinine, and total bilirubin), length of stay in the intensive care unit and surgical ward, the need for reintervention, and 90-day mortality.

Complications were categorized using the Clavien-Dindo classification [21] and summarized using the Comprehensive Complication Index [22]. Histopathological analysis of the explanted liver included number and size of nodules, tumor grade, vitality percentage, and the presence of satellitosis, pathological macrovascular, and microvascular invasion.

Post-transplant follow-up was standardized across both centers. Patients were monitored regularly in outpatient clinics and underwent contrast-enhanced computed tomography every 6 months for the first 5 years or earlier in the presence of suspected recurrence. Tumor recurrence was defined based on histologic confirmation or radiologic findings discussed by a multidisciplinary team.

### Statistical analysis

Qualitative variables were expressed as frequencies and percentages and quantitative variables as means and standard deviation (SD), if normally distributed and as medians and interquartile range (IQR) otherwise. Comparison of means between groups was done by Student’s *t*-test (pooled *t*-test) or with the nonparametric Mann–Whitney. To compare percentages between groups, a contingency table analysis was used with the chi-square test or Fisher’s exact test when the frequency of events was low. OS, DFS, and GS were estimated using the Kaplan–Meier method and compared using the log-rank test.

To assess the impact of time from diagnosis to transplant, receiver operating characteristic (ROC) curves were generated, and optimal cut-offs were identified using the Youden index. A multivariable Cox proportional hazards model was constructed to identify the independent predictors of recurrence, including patient demographics, BCLC stage, type of locoregional therapy, and transplant strategy. All statistical analyses were performed using R version 3.3.2 and RStudio version 1.0.44, with the graphical user interface provided by rBiostatistics.com.

## Results

### Study population

During the study period, a total of 486 LT for HCC were performed across the two centers. Among them, 182 patients (37%) underwent LDLT and 304 patients (63%) received DDLT. KFSHRC accounted for 244 procedures, including all LDLTs and 62 DDLTs, while UNIMORE performed 242 DDLTs.

Among the 182 LDLTs performed, 158 involved right hemiliver grafts without the middle hepatic vein (MHV), in which venous drainage of segment V and/or VIII was reconstructed when appropriate; 21 were left hemiliver grafts including the MHV; 1 was a dual graft; and 2 were whole liver grafts procured in the context of domino liver transplantation. Graft selection was based on donor anatomy and volumetric criteria, following institutional protocols.

Baseline characteristics of the two cohorts are summarized in Table 1.

**Table 1:** Descriptive statistics of study population

	LDLT (*n* = 182)	DDLT (*n* = 304)	*p*-value
Age	60.4 ± 7.9	58.6 ± 8.1	< 0.05
Male	126 (69.2)	246 (80.9)	< 0.05
BMI, kg/m^2^	27.3 ± 4.5	26.4 ± 4.0	< 0.05
Charlson Comorbidity Index	7 ± 1	7 ± 1	0.71
Admission to ICU before LT	2 (1.1)	10 (3.3)	0.22
Etiology of liver disease			< 0.05
Alcohol	3 (1.6)	26 (8.6)	
HBV	61 (33.5)	68 (22.4)	
HCV	54 (29.7)	159 (52.3)	
NASH	36 (19.8)	29 (9.5)	
Other	28 (15.4)	22 (7.2)	
Portal vein thrombosis	18 (9.9)	41 (13.5)	0.30
MELD	12.7 ± 5.9	13.4 ± 7.7	0.29
Presence of esophageal varices	93 (51.1)	172 (56.6)	0.28
HCC features			
Incidental at LT	15 (8.2)	7 (2.3)	< 0.05
N° of nodules	1.6 ± 0.9	1.7 ± 1.1	0.67
Size of biggest nodule, mm	29.4 ± 16.1	26.6 ± 14.7	0.24
Milan criteria IN	132 (72.5)	242 (79.6)	0.13
Up to seven criteria IN	158 (86.8)	270 (88.8)	0.55
BCLC stage			0.24
BCLC 0	40 (22.0)	69 (22.7)	
BCLC A	101 (55.5)	182 (59.9)	
BCLC B	39 (21.4)	52 (17.1)	
BCLC C	2 (1.1)	1 (0.3)	
Major vascular invasion contact to major vessels	7 (3.8)	4 (1.3)	0.08
AFP at diagnosis ng/ml	7.0 (23.15)	7.6 (17.4)	0.41
AFP at LT, ng/ml	5.9 (14.7)	5.4 (15.2)	0.15

Data are given as *n* (%) and median (IQR) or mean ± SD

*BMI* body mass index; *HBV* Hepatitis B virus; *HCV* Hepatitis C virus; *NASH* Nonalcoholic steatohepatitis *AFP* Alfafetoprotein

A significantly higher proportion of male patients was observed in the DDLT group (80.9% vs. 69.2%, *p* < 0.05). Patients in the LDLT group presented a slightly higher mean body mass index (BMI) (27.3 ± 4.5 vs. 26.4 ± 4.0, *p* = 0.02). No significant differences were observed in comorbidity burden as assessed by the Charlson comorbidity index or in the prevalence of portal vein thrombosis, esophageal varices, or admission to intensive care prior to transplantation.

Underlying liver disease differed significantly between groups. The DDLT group had a higher prevalence of alcohol-related and HCV-related liver disease, whereas the LDLT group had more cases of HBV-related and NASH-related disease.

Incidental hepatocellular carcinoma was more frequently found in LDLT recipients (8.2% vs. 2.3%, *p* = 0.005), while no statistically significant differences were detected regarding the number of nodules, maximum tumor diameter, or inclusion within Milan or up-to-seven criteria. BCLC staging and AFP levels at diagnosis and at transplant were also comparable between the two groups.

### Intraoperative outcomes

Intraoperative variables are reported in Table 2.

**Table 2:** intraoperative and perioperative outcomes of LT procedures

	LDLT (*n* = 182)	DDLT (*n* = 304)	*p*-value
*Intraoperative data*			
EBL (ml)	2394.8 ± 3205.3	2148.7 ± 2386.3	0.40
OT (min)	433.7 ± 108.2	434.9 ± 123.2	0.92
pRBC transfused units	5.0 ± 7.5	4.1 ± 4.7	0.18
Patients transfused with FFP (%)	110 (60.4)	112 (36.8)	< 0.05
Patients transfused with platelets (%)	92 (55.6)	75 (24.7)	< 0.05
Reperfusion syndrome	9 (4.9)	51 (16.8)	< 0.05
Caval reconstruction			< 0.05
Piggyback	182 (100)	272 (89.5)	
Caval replacement	0 (0)	32 (10.5)	
Arterial conduit	5 (2.7)	4 (1.3)	0.45
Biliary reconstruction			< 0.05
Hepaticojejunostomy	37 (20.3)	36 (12.0)	
Duct-to-duct anastomosis	145 (79.7)	268 (88.0)	
*Postoperative data*			
AST postoperative day 5, UI/l	67.2 ± 63.9	91.9 ± 88.1	< 0.05
ALT postoperative day 5, UI/l	151.1 ± 143.5	306.1 ± 301.0	< 0.05
INR postoperative day 5	1.6 ± 0.4	1.3 ± 0.3	< 0.05
Creatinine postoperative day 5, mg/dl	1.2 ± 1.3	0.9 ± 0.6	0.22
Total bilirubin postoperative day 5, mg/dl	3.0 ± 2.2	3.5 ± 3.2	0.08
C-D (90 days)			< 0.05
0	43 (23.6)	104 (34.2)	
I	16 (8.8)	32 (10.5)	
II	46 (25.3)	49 (16.1)	
IIIa	35 (19.2)	29 (9.5)	
IIIb	16 (8.8)	26 (8.6)	
IVa	12 (6.6)	23 (7.6)	
IVb	8 (4.4)	19 (6.3)	
V	6 (3.3)	22 (7.2)	
Low-grade morbidity (C-D ≤ II)	62 (34.1)	81 (26.6)	0.08
CCI (90 days)	26.2 ± 23.4	26.2 ± 29.1	0.99
ICU stay, days	6.2 ± 9.0	5.7 ± 13.4	0.72
LHS	24.9 ± 20.9	22.7 ± 21.9	0.06
Reintervention (90 days)	24 (13.2)	42 (13.8)	0.84

Data are given as *n* (%) and median (IQR) or mean ± SD

*EBL* estimated blood loss; *OT* operative time; *pRBC* packed red blood cells; *FFP* Fresh frozen plasma; *AST* aspartate aminotransferase (UI/l); *ALT* Alanine aminotransferase (UI/l); *INR* international normalized ratio; *C-D* Clavien-Dindo classification of surgical complications; *CCI* comprehensive complication index; *ICU* intensive care unit; *LHS* Length of hospital stay

The two groups showed similar OT (433.7 ± 108.2 vs. 434.9 ± 123.1 min, *p* = 0.92) and EBL (2394.8 ± 3205.3 vs. 2148.7 ± 2386.3 mL, *p* = 0.40). The number of pRBC units transfused did not differ significantly, although a higher proportion of LDLT recipients received FFP and platelet transfusions (*p* < 0.05 for both).

Reperfusion syndrome occurred more frequently in the DDLT group (16.8% vs. 4.9%, *p* < 0.05). Caval reconstruction was performed using the piggyback technique in all LDLT cases, whereas 10.5% of DDLT recipients required caval replacement (*p* < 0.05). The use of arterial conduits was similar. Hepaticojejunostomy was more common in the LDLT group (20.3% vs. 12.0%, *p* = 0.02).

### Postoperative outcomes

Postoperative laboratory values on day five showed higher transaminase levels and INR in the DDLT group, although the clinical significance of these differences is likely limited. Creatinine and total bilirubin levels were comparable.

The overall distribution of complications according to the Clavien-Dindo classification is detailed in Table 2. A higher incidence of grade IIIa complications was observed in LDLT recipients, consistent with the increased technical complexity of the procedure, particularly regarding biliary reconstruction. However, the rates of reintervention, length of ICU stay, hospital stay, and 90-day mortality were not statistically different between groups.

The 90-day mortality rate was numerically higher in the DDLT cohort (7.2% vs. 3.3%, *p* = 0.07), though this difference did not reach statistical significance. The overall postoperative burden of complications, as assessed by the CCI, was similar in both groups.

### Explant pathology

The histological findings of the explanted livers are summarized in Table 3. The average number of tumor nodules was comparable between groups, although the LDLT group presented a significantly larger maximum nodule diameter (30.4 ± 17.8 mm vs. 26.6 ± 24.6 mm, *p* < 0.05) and a higher percentage of tumor vitality (69.6% vs. 49.8%, *p* < 0.05). Satellitosis was less frequent among LDLT recipients (3.9% vs. 10.2%, *p* < 0.05). No significant differences were found in the rates of pathological microvascular or macrovascular invasion. Tumor grading, available in 151 LDLT and 270 DDLT cases, revealed a higher prevalence of poorly differentiated tumors in the DDLT group.

**Table 3:** Details of explant pathology

	LDLT (*n* = 182)	DDLT (*n* = 304)	*p*-value
Number of nodules	1.8 ± 1.1	2 ± 1.9	0.32
Size of biggest nodule, mm	30.4 ± 17.8	26.6 ± 24.6	< 0.05
Vitality, %	69.6 ± 38.9	49.8 ± 42.8	< 0.05
Satellitosis	7 (3.8)	29 (9.5)	< 0.05
Pathological macrovascular infiltration	5 (2.7)	5 (1.6)	0.46
Pathological microvascular infiltration	28 (15.4)	37 (12.2)	0.45
Grading (values on 151 LDLTs and 270 DDLTs)			< 0.05
Gx	0 (0)	66 (24.4)	
G1	48 (31.8)	31 (11.5)	
G2	83 (55.0)	114 (42.2)	
G3	16 (10.6)	57 (21.2)	
G4	4 (2.6)	2 (0.7)	

Data are given as *n* (%) and median (IQR) or mean ± SD

### Survival and recurrence

**Fig. 1:**
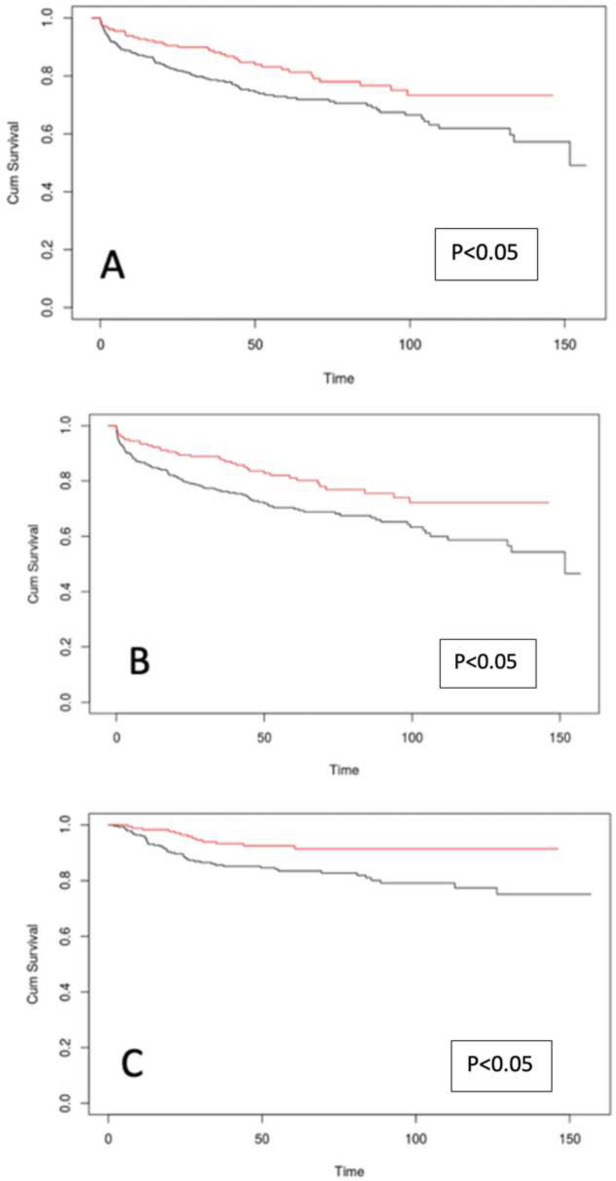
Overall survival (**A**), disease-free survival (**B**) and graft survival (**C**) of patients undergoing LDLT (red line) versus DDLT (black line)

Median follow-up duration was 55.13. Survival outcomes are depicted in Fig. 1.

The LDLT group showed superior OS at 1, 5, and 10 years (93, 82, and 73%, respectively) compared to the DDLT group (88, 73, and 62%, *p* < 0.05).

DFS rates were also significantly better in the LDLT group at the same timepoints (98, 93, and 91% vs. 95, 83, and 77%, *p* < 0.05).

**Fig. 2:**
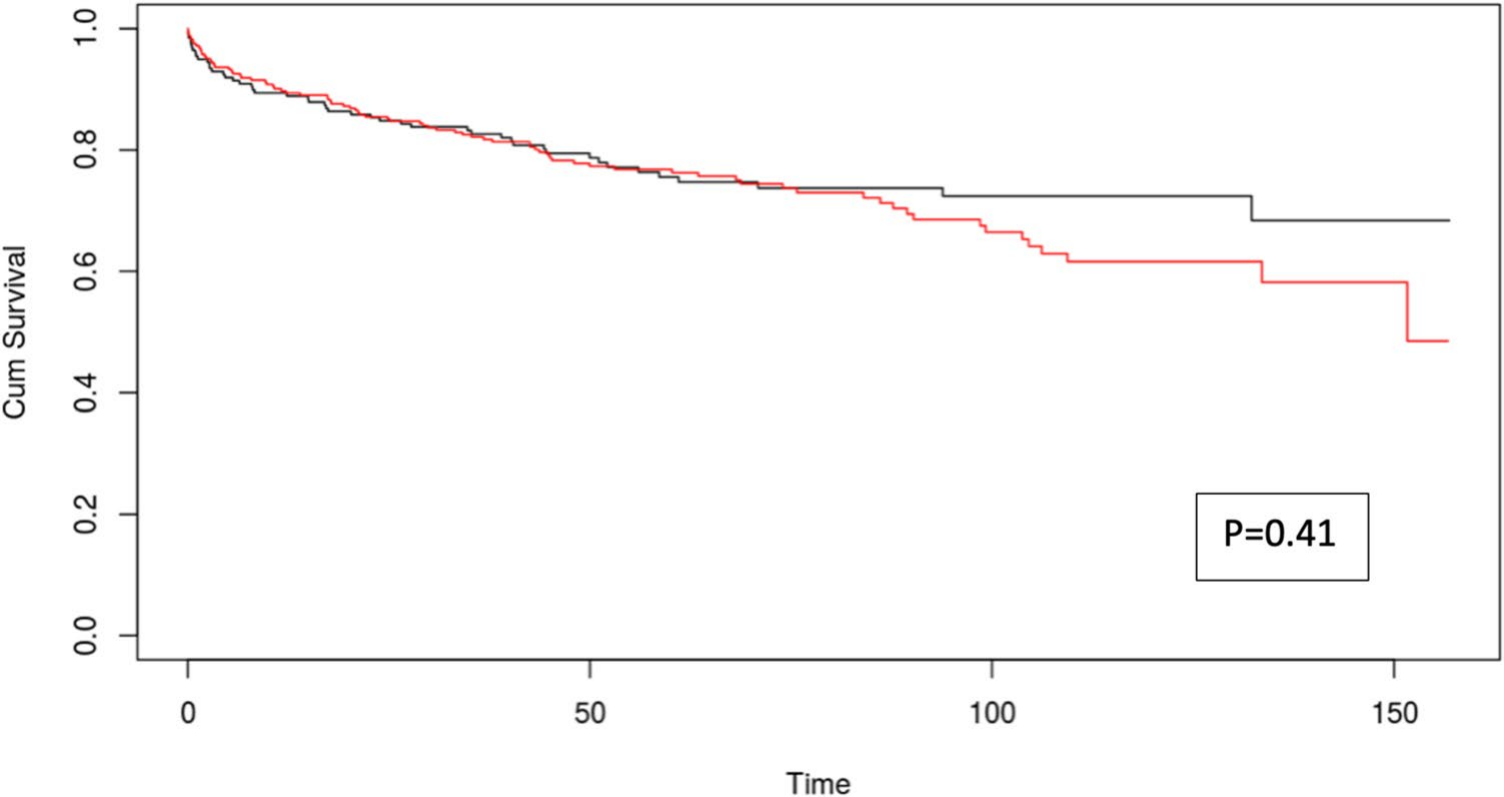
Kaplan–Meier curves for overall survival of patients undergoing liver transplant before (black line) and after (red line) 235 days from diagnosis

GS followed a similar trend, with higher rates observed in the LDLT cohort (*p* < 0.05) (Fig. 2).

### Timing of transplantation

The interval between diagnosis and transplantation was significantly shorter in the LDLT group (median 186 days) compared to the DDLT group (median 410 days, *p* < 0.05). Downstaging and bridging procedures were more frequently performed in the DDLT group (84% vs. 50%, *p* < 0.05), reflecting the longer wait times and need for tumor control while on the list.

**Fig. 3:**
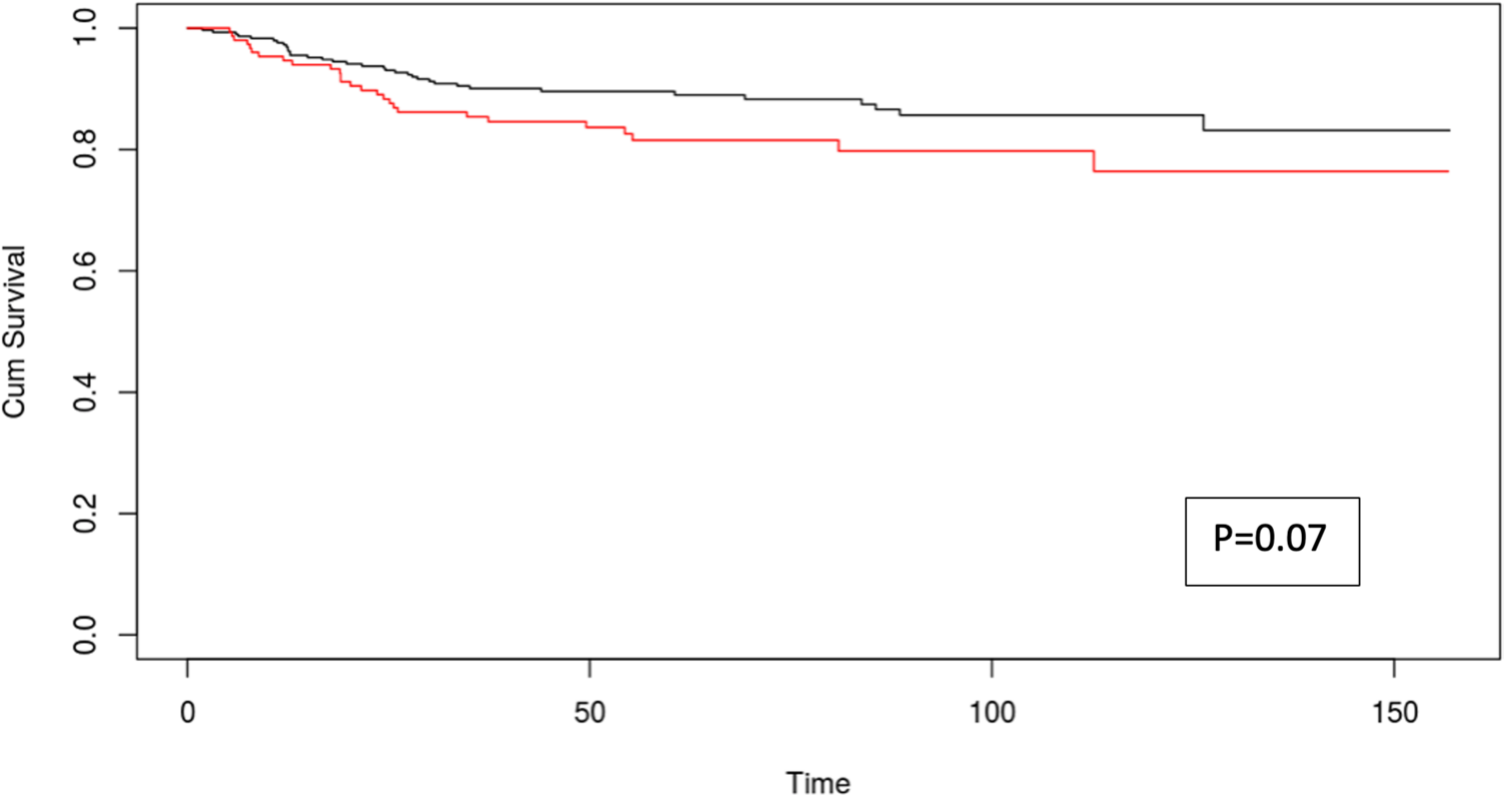
Kaplan–Meier curves for disease free survival of patients undergoing liver transplant before (black line) and after (red line) 476 days from diagnosis

ROC curve analysis identified 235 days as the optimal cut-off for overall survival (AUC 0.512) and 476 days for disease-free survival (AUC 0.568). Kaplan–Meier curves stratified by these timepoints showed no significant difference in overall survival (*p* = 0.41, Fig. 2), while a trend toward increased recurrence was observed in patients waiting more than 476 days (*p* = 0.07, Fig. 3).

### Multivariable analysis

A multivariable Cox regression model was constructed to identify predictors of tumor recurrence. After adjusting for age, sex, BCLC stage, and locoregional therapy, two independent predictors of recurrence were identified: BCLC stage B or C (HR 1.98; 95% CI 1.14–3.46, *p* = 0.017) and undergoing DDLT rather than LDLT (HR 3.03; 95% CI 1.36–5.08, *p* = 0.017).

Although BCLC stage C is generally considered a contraindication to transplantation due to the association with macrovascular invasion, in our series, three patients initially classified as BCLC C due to type 2 portal vein tumor thrombus were successfully downstaged and transplanted. In the multivariable model, BCLC B and C stages were grouped to reflect intermediate-to-advanced tumor burden at listing.

## Discussion

This study compared long-term outcomes in patients with HCC undergoing either LDLT or DDLT at two high-volume institutions. Our findings indicate that LDLT is associated with significantly better OS, DFS, and GS. Moreover, the LDLT strategy emerged as an independent protective factor against HCC recurrence in multivariable analysis. These results support the increasing recognition of LDLT as a valid and, in selected cases, superior therapeutic approach for transplantable HCC.

Baseline characteristics were largely comparable between the two groups, with no significant differences in tumor burden, vascular invasion, or MELD score. While patients undergoing LDLT showed higher vitality and slightly larger tumors at explant pathology, this did not translate into inferior outcomes. On the contrary, LDLT recipients demonstrated a lower recurrence rate and improved long-term survival. These results are in line with prior literature suggesting that early transplantation, achievable through LDLT, can minimize the impact of tumor progression and biologic aggressiveness.

At the time of diagnosis, patients with HCC stand at a therapeutic crossroads, where the availability of a living donor may significantly influence the overall treatment strategy. When LDLT is feasible, it allows for timely access to curative transplantation, often minimizing or even avoiding the need for downstaging or bridging therapies. This approach reduces both waiting time and the risk of disease progression.

Conversely, when LDLT is not available, patients are typically listed for DDLT and may face prolonged waiting times. In this scenario, locoregional treatments, surgical resection, or even systemic therapies are often employed to maintain transplant eligibility and control disease progression. Several studies have demonstrated that these modalities, when applied appropriately, can lead to excellent long-term outcomes and should be viewed as integral components of a multimodal treatment strategy [23, 24].

A major difference between the two groups was the time interval from diagnosis to transplantation. LDLT allowed for significantly shorter waiting times, reducing the need for downstaging or bridging therapies and potentially minimizing the risk of dropout. Although the statistical analysis did not confirm a significant association between longer waiting time and overall survival, a trend toward higher recurrence was observed in patients waiting more than 476 days. These findings support the concept of “time sensitivity” in the management of HCC, as delays may provide the opportunity for tumor progression, particularly in patients with aggressive tumor biology.

At the same time, the absence of a significant difference in survival between patients undergoing early versus late transplantation suggests that current downstaging and bridging strategies are effective in selected cases. Additionally, longer waiting times may help to identify biologically aggressive tumors that are unlikely to benefit from transplantation, thereby functioning as a “test of time.”

This dual role of timing—both as a modifiable variable and as a surrogate for tumor biology—requires careful balancing in clinical decision-making.

The improved disease-free and overall survival associated with LDLT persisted even after adjusting for waiting time, tumor stage, and locoregional treatments. This raises the possibility that other factors intrinsic to the LDLT strategy may play a role.

One hypothesis is that LDLT allows for better patient selection and optimization based on tumor response, liver function, and patient fitness.

Additionally, the quality of living donor grafts, which typically come from younger and healthier donors with minimal ischemia time, may contribute to improved immunologic and oncologic outcomes, including lower recurrence risk [25, 26].

Moreover, the lower degree of ischemia–reperfusion injury observed in patients undergoing LDLT, as reflected by significantly lower postoperative AST and ALT levels, may represent an additional factor contributing to the reduced incidence of HCC recurrence in this group. This hypothesis is supported by previous studies, which have linked milder reperfusion injury to improved oncologic outcomes after liver transplantation [27].

Finally, unmeasured differences in tumor biology between groups cannot be excluded. It is plausible that patients who proceed to LDLT represent a biologically more favorable cohort, either due to better response to therapy or lack of aggressive features at presentation. These elements collectively may explain why LDLT is independently associated with lower recurrence, beyond the sole effect of shorter wait times.

Postoperative outcomes were comparable between LDLT and DDLT recipients, with similar overall complication rates. However, patients in the LDLT group experienced a higher incidence of grade IIIa complications, a finding that likely reflects the inherent technical complexity of the procedure. In particular, the greater difficulty of the reconstruction phase in LDLT—especially in terms of biliary anatomy—may increase the need for interventional radiologic or endoscopic procedures to manage biliary complications [28].

Our findings underscore the clinical value of LDLT not only for individual patients, but also for the transplant system as a whole. Expanding LDLT programs can alleviate pressure on deceased donor waiting lists, increase the overall number of transplants, and offer timely curative treatment to patients at risk of progression [29].

Moreover, the implementation of minimally invasive donor hepatectomy techniques may further improve the safety and acceptance of living donation. These developments should encourage wider dissemination of LDLT in Western countries, where it remains underutilized compared to Asia.

This study is subject to several limitations. First, its retrospective design may be associated with unrecognized selection biases, despite the use of prospectively maintained databases and the inclusion of consecutive cases. Second, although the two institutions applied similar selection criteria, practice patterns regarding pre-LT treatments and donor availability may differ.

We acknowledge that recipient populations differed geographically and culturally, as the LDLT group included only patients from the Middle East, while the DDLT group included a predominantly European cohort. Such differences may reflect not only sociocultural factors but also diverse etiologies of cirrhosis—HBV being more prevalent in Arab populations while alcohol-related liver disease and HCV are predominant in this cohort. These distinct pathogenetic patterns may have influenced tumor biology, background liver disease, and response to pre-transplant therapies. Although these variables were not the primary focus of the present analysis, we recognize this heterogeneity as a potential limitation when interpreting comparative oncologic outcomes between groups.

A possible limitation of the study is the lack of detailed donor characteristics, such as age, gender, and BMI, which were not uniformly available in our database. Although both institutions adopt standardized donor assessment criteria, these missing data prevent adjustment for potential differences in graft quality. We acknowledge this as a limitation in interpreting outcome differences across transplant types.

## Conclusion

This bi-institutional analysis confirms that LDLT offers superior long-term outcomes for patients with HCC compared to DDLT. LDLT was independently associated with improved OS and DFS, as well as reduced recurrence risk, regardless of tumor stage and timing of transplant.

This consistent advantage reinforces the role of LDLT as a fundamental component of the therapeutic strategy for HCC patients requiring liver transplantation and supports its broader implementation in transplant programs.
